# Clinical and genotypic analysis in determining dystonia non-motor phenotypic heterogeneity: a UK Biobank study

**DOI:** 10.1007/s00415-022-11307-4

**Published:** 2022-08-04

**Authors:** Megan E. Wadon, Eilidh Fenner, Kimberley M. Kendall, Grace A. Bailey, Cynthia Sandor, Elliott Rees, Kathryn J. Peall

**Affiliations:** 1 Neuroscience and Mental Health Research Institute, Division of Psychological Medicine and Clinical Neurosciences, Cardiff University, Hadyn Ellis Building, Maindy Road, Cardiff, UK; 2 MRC Centre for Neuropsychiatric Genetics and Genomics, Division of Psychological Medicine and Clinical Neurosciences, Cardiff University, Cardiff, UK; 3 UK Dementia Research Institute, Cardiff University, Cardiff, UK

**Keywords:** Dystonia, Psychiatric, Pain, Sleep, Cognition

## Abstract

The spectrum of non-motor symptoms in dystonia remains unclear. Using UK Biobank data, we analysed clinical phenotypic and genetic information in the largest dystonia cohort reported to date. Case–control comparison of dystonia and matched control cohort was undertaken to identify domains (psychiatric, pain, sleep and cognition) of increased symptom burden in dystonia. Whole exome data were used to determine the rate and likely pathogenicity of variants in Mendelian inherited dystonia causing genes and linked to clinical data. Within the dystonia cohort, phenotypic and genetic single-nucleotide polymorphism (SNP) data were combined in a mixed model analysis to derive genetically informed phenotypic axes. A total of 1572 individuals with dystonia were identified, including cervical dystonia (*n* = 775), blepharospasm (*n* = 131), tremor (*n* = 488) and dystonia, unspecified (*n* = 154) groups. Phenotypic patterns highlighted a predominance of psychiatric symptoms (anxiety and depression), excess pain and sleep disturbance. Cognitive impairment was limited to prospective memory and fluid intelligence. Whole exome sequencing identified 798 loss of function variants in dystonia-linked genes, 67 missense variants (MPC > 3) and 305 other forms of non-synonymous variants (including inframe deletion, inframe insertion, stop loss and start loss variants). A single loss of function variant (*ANO3*) was identified in the dystonia cohort. Combined SNP and clinical data identified multiple genetically informed phenotypic axes with predominance of psychiatric, pain and sleep non-motor domains. An excess of psychiatric, pain and sleep symptoms were evident across all forms of dystonia. Combination with genetic data highlights phenotypic subgroups consistent with the heterogeneity observed in clinical practice.

**Supplementary Information:**

The online version contains supplementary material available at 10.1007/s00415-022-11307-4.

## Introduction

Dystonia is a hyperkinetic movement disorder resulting in abnormal posturing, pain, functional impairment and reduced quality of life [1]. Onset of motor symptoms may be in childhood (< 20 years) or adult-life (> 20 years), with muscle involvement being generalised (multiple body regions including the trunk), segmental (adjacent body regions), multifocal or focal (single body region). To date, > 30 Mendelian inherited, dystonia-causing genes with distinct motor phenotypes have been identified [2].

Dystonia is increasingly recognised to include both motor and non-motor symptoms, with much of the non-motor work to date focusing on psychiatric symptomatology. Anxiety and depression have been shown to be the most prominent features [3–6], impacting quality of life and motor symptom severity [7]. Other non-motor features described previously include pain, sleep disturbance and changes to cognitive function, with these being less well investigated, although pain has been reported in up to 88.9% of those with cervical dystonia, with a frequency up to five times higher than unaffected controls [6, 8]. Impaired sleep quality has also been reported in up to 77.3% of those with cervical dystonia, together with increased rates of excessive daytime sleepiness and fatigue.[5, 6, 9–11]. Sleep disturbance has been linked to higher levels of anxiety, depression and pain, suggesting interplay between these multiple non-motor traits [11, 12]. Results of cognitive assessments in dystonia cohorts have been conflicting, with suggestion of variation between dystonia subtypes [13–15]. Several studies have noted impaired prospective memory, processing speed, memory recall, working memory and verbal memory in those with cervical dystonia, while others have found normal verbal memory and executive function [14–16]. Those with blepharospasm appear to demonstrate fewer areas of cognitive deficit, although some studies have described impaired overall cognitive function [16–18].

Prospective recruitment and detailed phenotypic assessment of cohorts represent a lengthy and resource demanding process, often reliant of participant recall for details of past diagnoses and potential loss to future follow-up. In recent years, the anonymous linking of clinical data together with large, population-level clinical, genetic and imaging data collection provides an invaluable tool for the investigation of multiple clinical disorders [19]. The UK Biobank (UKBB) represents one such resource having recruited over half a million participants and consisting of data from hospital admissions, self-reported clinical and symptomatic information, primary care records, genetic and imaging data. This study derives dystonia and matched control cohorts from the UKBB, using a previously published clinically validated algorithm [20], with subsequent determination of dystonia non-motor symptom characteristics, the rate of variants in known dystonia-causing genes and the potential role of genotype in influencing non-motor phenotypic variation.

## Methods

### Participants

Participants were recruited to the UKBB, a prospective health study of > 500,000 individuals located across the UK, between 2006 and 2010 [21, 22]. UKBB is approved by the Research Ethics Committee (reference 16/NW/0274), with data released to Cardiff University following application (study application code: 13310) access to this data is available through application to the UK Biobank. The International Classification of Disorders (ICD)-10 system (field codes: 41202, 41204 and 41270) and Primary Care (General Practice/Family Doctor) records (Read codes v2) were used to identify those diagnosed with dystonia (Supplementary Table 1), using a previously reported algorithm demonstrating a 79% sensitivity for identification of those diagnosed with dystonia [20]. Participants were excluded if they were reported to have been diagnosed with drug-induced dystonia, any other movement disorder or related condition (Supplementary Table 2), with the aim of excluding potential diagnoses of secondary dystonia. Individual dystonia diagnostic sub-groups with < 100 recorded participants were not included for onward analysis. A control cohort, matched for age and sex, was randomly generated from the remaining cohort on a 15: 1 (control: case) ratio.

### Assessment of non-motor symptoms

#### Psychiatric symptoms

Psychiatric symptoms were determined as has been described in previous UKBB studies [23]. These included: (i) documentation of an ICD-10 diagnosis within the “Mental and Behavioural Disorders” section (field codes: 41202 or 41204), (ii) individuals reviewed by a GP (field code: 2090) or psychiatrist (field code: 2100) for anxiety, tension, or depression., (iii) self-reported psychiatric symptoms (field codes: 1930, 1940, 1960, 1970, 1980, 1990, 2000, 2010, 2030) which had a completion rate of > 93% for each question within the dystonia cohort. We also identified individuals with psychiatric conditions using Primary Care records (Read Code v2). Each psychiatric diagnosis was considered independently, i.e. if a participant was linked with diagnostic codes for both depression and anxiety, they were included in both diagnostic categories. Although other methods of determining psychiatric symptoms are available within the UKBB, the proportion of those diagnosed with dystonia with a complete dataset was limited (e.g. completion rate for the Mental Health Questionnaire was 437/1572, < 30%) and therefore not included in this analysis.

#### Pain

Consistent with previous studies examining pain symptoms within the UK Biobank [24, 25], we determined the presence of pain by responses given to standardised questionnaires asking whether pain was present, or not, in one or multiple body regions in the last month (field code: 6159). Body regions included were headache, facial pain, neck or shoulder pain, back pain, stomach or abdominal pain, hip pain, knee pain or “pain all over”.

#### Sleep

As used previously for the analysis of sleep disturbance in UKBB participants, five aspects of sleep were analysed through use of a standardised questionnaire, with each being categorised as either high or low risk of sleep disturbance [26, 27]: (i) Insomnia (field code: 1200): trouble falling asleep at night or waking in the night (never/rarely = low risk, sometimes/usually = high risk), (ii) chronotype (field code: 1180): being a morning or evening person (early chronotype = low risk, late chronotype = high risk), (iii) sleep duration (field code: 1160): total reported hours slept in 24 h (7–8 h sleep = low risk, < 7 h or > 8 = high risk), (iv) snoring (field code:1210): participants reporting snoring = high risk, (v) daytime sleepiness (field code: 1220): falling asleep during the day (“never/rarely” or “sometimes” = low risk, “often” = high risk). A healthy sleep score was derived with participants gaining a point for each low-risk outcome and defined as ‘healthy sleep pattern (≥ 4), ‘intermediate sleep pattern (2 or 3) and ‘poor sleep pattern’ (≤ 1).

#### Cognition

Cognition was assessed using both touch screen devices and online tests. Both testing mediums assessed the same domains, and therefore, we chose those completed using a touch screen device for consistency and given its higher rate of completion amongst the dystonia cohort (99.7% vs. 19.3%). Four domains of testing were included: (i) numeric memory (field code: 4282)—maximum number of digits remembered correctly, (ii) prospective memory (field code: 20018)—subdivided into “Good recall” (correct recall attempt), “intermediate recall” (correct second attempt) and “bad recall” (incorrect/no recall), (iii) fluid intelligence (field code: 20016)—number of fluid intelligence questions answered correctly, (iv) pairs matching (field code: 20132)—incorrect matches made for each round [28].

### Analysis of whole exome sequencing data for variants in Mendelian inherited genes linked with dystonia

The presence of variants in Mendelian inherited genes linked with dystonia was determined by analysis of the plink project files generated from the whole exome sequencing data available in the first UKBB release (*n* = 200,643) [29]. Briefly, exome data were analysed in Hail, [30] with samples or variants excluded if they had a call rate < 95% and an allele count > 5. Variants were annotated with their functional consequence using the variant effect predictor (version 104.3) function in Hail. Additional variant annotations from dbNSFP (version 4.1a) were annotated using Hail [31]. Analysis was restricted to 32 genes that have previously been associated with dystonia [32]. Individuals with synonymous, missense, loss of function (splice acceptor, splice donor, stop gain and frameshift) and remaining forms of non-synonymous (inframe deletion, inframe insertion, stop loss and start loss) variants were identified.

Missense variants were classified as to their likelihood of pathogenicity using a composite score of ‘missense badness’, PolyPhen-2 and constraint (MPC) pathogenicity classifier, which has previously been shown to have a high specificity for pathogenicity, with increased scores indicating a higher likelihood of deleteriousness [33]. We determined three MPC categories of increasing deleteriousness: MPC < 2, 2 ≤ MPC < 3, MPC ≥ 3. Variants were classified as either singleton (present once in the dataset) or rare (present 2–5 times in the dataset). The overall cohort was examined for both relatedness and ancestry, removing those indicated to be closer than third-degree relatives and where the principal components were > 3 standard deviations from the mean of individuals reporting their ethnic background as white. ClinVar (https://www.ncbi.nlm.nih.gov/clinvar/) databases were used to determine those variants that have been reported previously, their likelihood of pathogenicity and the clinical disorders with which they had been linked.

### Bayesian multiple phenotype mixed model analysis (BMPMM) to determine evidence for variation in clinical phenotypic axes

One of the challenges in dystonia is to understand the varying clinical presentation between individuals. This clinical heterogeneity suggests the existence of varying molecular aetiologies with different subtypes and influences. The classical approach to characterise clinical heterogeneity is to classify patients into discrete phenotypic subgroups, each displaying a characteristic set of symptoms. Although this approach is appealing, there are many shortfalls including arbitrary choice of the clinical variables. Additionally, the use of discrete groups also limits the comparison of phenotypes to binary tests of absent/present or mild/severe, not reflecting the continual nature of phenotypic variability. Analysis of continuous traits, as we have used here, has greater statistical power and is more biologically relevant, allowing us to examine all individuals regardless of the frequency of their particular phenotypic presentation. The approach is based on PHENIX (PHENotype Imputation eXpediated), a sensitive approach exploiting genetic relationships to impute missing phenotypes, but that can also be used for genetically guided dimensionality reduction in multiple traits. Here we model phenotypes as a combination of genetic and environmental factors, producing multiple phenotypic axes, each representing a continuous pattern of variation between multiple co-varying phenotypes.

This analysis made use of the single-nucleotide polymorphism (SNP) genotype data provided by the UKBB, with participants only included if they had both genotypic and phenotypic data [21]. A kinship matrix was created using Genome-wide Efficient Mixed Model Association (GEMMA) (version 0.98.4) from SNP data [34–36]. Kinship and phenotypic matrices were combined, using a Bayesian low-rank matrix factorisation model, to create phenotypic axes using the PHENIX package in R [37]. The resulting latent variables constitute phenotypic axes representing the severity of multiple non-independent clinical phenotypes. Variables with ≥ 10% of data missing (e.g. > 1/10 individuals had not completed that test/question) were removed when determining the phenotypic axes and then reintroduced in identifying the clinical relationships within the predetermined axes. Variables that showed little variation within each cohort (e.g. minimal participants had reported the symptom) were removed from this analysis. Analysis was undertaken in the overall dystonia cohort and any of the individual subgroups where genotype data were available for a minimum of 400 participants.

### Statistical analysis

Categorical data were compared using chi-squared tests or Fisher’s exact tests where appropriate. Continuous data were checked for normality, before being compared using Independent 2-group Mann–Whitney *U* tests. Comparisons were made between the complete dystonia cohort and control group, as well as each dystonia subtype and the control cohort. All *p* values were adjusted for multiple comparisons using a Bonferroni correction (significance threshold *p* < 0.05).

## Results

### Participants

One thousand, five hundred and seventy-two individuals (989 female, 583 male), mean age of 59 years (SD: 8.1, range: 40–70), diagnosed with dystonia were identified, with 24,012 (14,889 female, 9123 male) forming the control group matched for both age (*p* = 0.789) and gender (*p* = 0.471, Table 1). Using the combined ICD-10 and Primary Care diagnostic groups (Supplementary Table 1), only cervical dystonia, blepharospasm, dystonia with tremor and dystonia unspecified subgroups met our criteria for onward analysis (*n* ≥ 100).

**Table 1:** Demographic characteristics of the identified dystonia and control cohorts

Diagnosis	*N*	Age			Gender	
		Mean	Standard deviation	Range	Male	Female
Dystonia	1572	59	8.1	40–70	583	989
Idiopathic familial dystonia	3	66	3.5	62–68	1	2
Idiopathic non-familial dystonia	1	58	–	–	0	1
Cervical dystonia	775	55.8	8.1	40–70	283	492
Idiopathic orofacial dystonia	6	47.7	8.8	40–64	3	3
Blepharospasm	131	60.4	7.3	42–70	40	91
Idiopathic torsion dystonia	1	47	–	–	0	1
Writer’s cramp	4	53.8	2.5	51–57	1	3
Myoclonic dystonia	1	61	–	–	1	0
Dystonia with tremor	488	59.6	7.7	40–70	201	287
Other dystonia	8	54.9	5.9	47–65	2	6
Dystonia unspecified	154	57.5	8.1	41–70	51	103
Control	24,012	59	8.1	40–70	9123	14,889

### Non-motor symptom phenotypic analysis

All statistical comparisons reported below are between the overall dystonia cohort, or individual dystonia diagnostic groups, and the control group.

#### Psychiatric symptoms

As observed in multiple previous UK population-level studies of neurological disorders, rates of recorded diagnoses were higher in the primary care dataset, compared to that of the hospital record-derived ICD-10 [38]. Here, significantly higher rates of multiple psychiatric disorders were observed across the dystonia cohort, including anxiety (*p* < 2.2 × 10^–16^), depression (*p* < 2.2 × 10^–16^), eating disorders (*p* = 0.002), serious mental illness (*p* = 0.002) and substance use disorder (*p* < 2.2 × 10^–16^), with the same pattern observed in cervical dystonia and dystonic tremor groups (Table 2). By contrast, those with blepharospasm were reported to have significantly higher rates of anxiety (*p* = 0.002) and depression (*p* = 0.002), while those with unspecified forms of dystonia demonstrated only higher rates of serious mental illness (*p* = 0.004). Results from the symptom reporting questionnaires identified a significantly higher number of the dystonia group reporting mood related symptoms across all examined domains, with exception of irritability and worry following embarrassment (Table 2). Those diagnosed with dystonia were also more likely to have been reviewed by a GP or psychiatrist for nerves, anxiety, tension or depression (*p* = 1.27 × 10^–10^ and *p* = 7.05 × 10^–14^, respectively). Individuals diagnosed with dystonic tremor demonstrated a similar symptom pattern to the overall dystonia cohort, while those diagnosed with cervical dystonia described a more specific pattern of symptoms, including higher rates of feeling tense (*p* = 0.024), suffering from nerves (*p* = 0.020), feeling fed-up (*p* = 0.002), mood swings (*p* = 0.006), but were not significantly more likely to have been reviewed by a GP or psychiatrist (*p* = 0.071 and *p* = 0.069, respectively). Unspecified forms of dystonia noted higher rates of two symptoms groups: ‘being a nervous person’ (*p* = 0.002) and ‘suffering from nerves’ (*p* = 0.001), along with seeing a GP or psychiatrist (*p* = 0.028 and p = 1.76 × 10^–4^, respectively). The only psychiatric symptom observed at a higher rate within the blepharospasm cohort was that of ‘guilty feelings’ (*p* = 0.010), with no excess rate of GP or Psychiatrist consultation reported. ICD-10 psychiatric diagnostic rates were examined despite their overall low reported rates (Supplementary Table 3). Bipolar Affective Disorder unspecified was the only diagnosis significantly higher in the overall dystonia cohort (*p* = 0.002) and only Asperger’s syndrome (*p* = 0.003) in the blepharospasm group. Schizophrenia (*p* = 0.037), bipolar affective disorder (*p* = 0.044), recurrent depressive disorder (*p* = 0.005), harmful use of tobacco (*p* = 6.03 × 10^–4^) and stuttering (*p* = 0.008) were present at higher rates in the dystonia, unspecified cohort.

**Table 2:** Scores for primary care and symptomatic psychiatric components

		Dystonia		Cervical dystonia		Blepharospasm		Tremor		Dystonia, unspecified		Control
			*p* value		*p* value		*p* value		*p* value		*p* value	
Primary care diagnoses—psychiatric disorders												
ADHD		0	–	0	–	0	–	0	–	0	–	0
Anxiety		383 (24.4%)	< 2.2 × 10^–16^***	200 (25..8%)	< 2.2 × 10^–16^***	15 (11.5%)	0.002**	160 (32.8%)	< 2.2 × 10^–16^***	5 (3.2%)	1	518 (2.2%)
ASD		0	–	0	–	0	–	0	–	0	–	2 (0.008%)
Conduct disorders		0	–	0	–	0	–	0	–	0	–	1 (0.004%)
Depression		485 (30.9%)	< 2.2 × 10^–16^***	259 (33.4%)	< 2.2 × 10^–16^***	20 (15.3%)	0.002**	196 (40.2%)	< 2.2 × 10^–16^***	7 (4.5%)	0.876	692 (2.9%)
Eating disorders		20 (1.3%)	0.002**	12 (1.5%)	0.002**	1 (0.8%)	0.360	7 (1.4%)	0.002**	0	-	14 (0.05%)
Serious mental illness		38 (2.4%)	0.002**	12 (1.5%)	0.002**	2 (1.5%)	0.087	21 (4.3%)	0.002**	3 (1.9%)	0.004**	31 (0.01%)
Substance use disorder		74 (4.7%)	< 2.2 × 10^–16^***	32 (4.1%)	< 2.2 × 10^–16^***	4 (3.1%)	0.195	36 (7.4%)	< 2.2 × 10^–16^***	2 (1.3%)	1	215 (0.9%)
Psychiatric symptoms												
Nervous person		448/1510 (29.7%)	1.37 × 10^–5^***	205/748 (27.4%)	0.515	35/127 (27.6%)	1	148/466 (31.8%)	2.00 × 10^–3^***	55/146 (37.7%)	0.002**	5602/23278 (24.1%)
Worrying/anxious feelings		951/1520 (62.6%)	2.59 × 10^–3^***	465/752 (61.8%)	0.327	73/125 (58.4%)	1	301/470 (64.0%)	0.008**	98/149 (65.8%)	0.725	13,445/23314 (57.7%)
Feels tense/”Highly strung”		359/1488 (24.1%)	6.40 × 10^–9^***	164/738 (22.2%)	0.024*	28/123 (22.8%)	1	122/466 (26.2%)	3.76 × 10^–5^***	41/139 (29.5%)	0.056	4085/23096 (17.7%)
Worries too long after embarrassment		765/1485 (51.5%)	0.274	361/730 (49.5%)	1	66/123 (53.7%)	1	253/465 (54.4%)	0.151	78/145 (53.8%)	1	11,091/22919 (48.4%)
Suffers from nerves		417/1485 (28.1%)	5.54 × 10^–10^***	190/739 (25.7%)	0.020*	34/126 (27.0%)	1	143/457 (31.3%)	9.61 × 10^–7^***	47/142 (33.1%)	0.006**	4787/22992 (20.8%)
Irritability		419/1469 (28.5%)	1	194/721 (26.9%)	1	27/125 (21.6%)	1	148/458 (32.1%)	0.314	46/144 (31.9%)	1	6237/22894 (27.2%)
Miserableness		725/1537 (47.2%)	0.093	352/757 (42.9%)	1	51/128 (39.8%)	1	243/479 (50.7%)	0.029*	68/150 (45.3%)	1	10,274/23553 (43.6%)
Fed-up feelings		728/1529 (47.6%)	2.61 × 10^–7^***	358/759 (47.2%)	0.002**	53/126 (42.1%)	1	241/475 (50.7%)	7.31 × 10^–5^***	65/146 (44.5%)	1	9431/23403 (40.3%)
Guilty feelings		509/1513 (33.6%)	0.011*	249/749 (33.2%)	0.421	55/126 (43.7%)	0.010*	155/468 (33.1%)	1	48/148 (32.4%)	1	6888/23312 (29.5%)
Mood swings		780/1527 (51.1%)	3.69 × 10^–4^***	392/753 (52.1%)	0.006**	53/128 (41.4%)	1	253/476 (53.2%)	0.015*	71/147 (48.3%)	1	10,623/23329 (45.5%)
Loneliness/isolation		370/1529 (24.2%)	5.59 × 10^–6^***	163/754 (21.6%)	0.890	36/130 (27.7%)	0.193	126/474 (26.6%)	1.96 × 10^–4^***	40/149 (26.8%)	0.238	4448/23527 (18.9%)
Seen GP/doctor for nerves, anxiety, tension, or depression		676/1547 (43.7%)	1.26 × 10^–10^***	306/764 (40.1%)	0.071	53/127 (41.7%)	1	237/480 (49.4%)	1.72 × 10^–9^***	72/152 (47.4%)	0.028*	8348/23780 (35.1%)
Seen a psychiatrist for nerves, anxiety, tension, or depression		278/1558 (17.8%)	7.05 × 10^–14^***	114/769 (14.8%)	0.069	26/127 (20.5%)	0.033*	100/486 (20.6%)	1.30 × 10^–8^***	35/151 (23.2%)	1.76 × 10^–4^***	2741/23865 (11.5%)

*ADHD* attention deficit hyperactivity disorder, *GP* general practitioner

*p* values are all vs control and are represented as post-Bonferroni correction for multiple comparisons, *p < 0.05; **p < 0.01; ***p < 0.001

#### Pain

A significantly higher number of those diagnosed with dystonia reported experiencing pain (*p* = 2.29 × 10^–19^), with this replicated in the cervical dystonia (*p* = 7.09 × 10^–11^), dystonic tremor (*p* = 1.51 × 10^–6^) and dystonia, unspecified (*p* = 1.60 × 10^–6^) groups (Table 3). The body regions affected varied between dystonia diagnostic subgroups with higher rates of headache (*p* = 1.08 × 10^–6^) and neck/shoulder pain (*p* = 5.80 × 10^–11^) reported in the cervical dystonia group, while facial pain was more prominent in the blepharospasm group (*p* = 0.004). Dystonia with tremor and unspecified forms reported more generalised pain involvement with significantly higher rates of ‘pain all over’ (*p* = 0.004) (Table 3).

**Table 3:** Comparison of pain, sleep and cognition scores between dystonia and control cohorts

	Dystonia		Cervical dystonia		Blepharospasm		Tremor		Dystonia, unspecified		Control
	*n* (%)	*p* value	*n* (%)	*p* value	*n* (%)	*p* value	*n* (%)	*p* value	*n* (%)	*p* value	*n* (%)
Pain	1129/1565 (72.1%)	2.29 × 10^–19^***	560/774 (72.3%)	7.09 × 10^–11^***	86/131 (65.6%)	0.283	347/485 (71.5%)	1.51 × 10^–6^***	121/151 (80.1%)	1.60 × 10^–6^ ***	14,519/23911 (60.7%)
Headache	430/1565 (27.5%)	6.63 × 10^–10^***	220/774 (28.4%)	1.08 × 10^–6^***	29/131 (22.1%)	1	136/485 (28.0%)	5.54 × 10^–4^***	41/151 (27.2%)	0.458	4912/23911 (20.5%)
Facial pain	60/1565 (3.8%)	4.47 × 10^–7^***	18/774 (2.3%)	1	13/131 (9.9%)	0.004**	17/485 (3.5%)	0.094	11/151 (7.3%)	0.004**	439/23911 (1.8%)
Neck/shoulder pain	551/1565 (35.2%)	4.82 × 10^–23^***	268/774 (34.6%)	5.80 × 10^–11^***	37/131 (28.2%)	1	164/485 (33.8%)	3.84 × 10^–6^***	71/151 (47.0%)	4.50 × 10^–10^ ***	5701/23911 (23.8%)
Back pain	493/1565 (31.5%)	3.22 × 10^–6^***	230/774 (39.7%)	0.101	46/131 (35.1%)	0.143	163/485 (33.6%)	7.57 × 10^–4^***	49/151 (32.5%)	0.565	6137/23911 (25.7%)
Stomach/abdominal pain	201/1565 (12.8%)	4.66 × 10^–7^***	100/774 (12.9%)	6.50 × 10^–4^***	15/131 (11.5%)	1	59/485 (12.2%)	0.089	25/151 (16.6%)	0.010*	2094/23911 (8.8%)
Hip pain	260/1565 (16.6%)	6.33 × 10^–9^***	100/774 (12.9%)	1	26/131 (19.8%)	0.032*	95/485 (19.6%)	3.12 × 10^–7^***	35/151 (23.2%)	9.12 × 10^–5^ ***	2730/23911 (11.4%)
Knee pain	402/1565 (25.7%)	1.30 × 10^–3^***	196/774 (25.3%)	0.119	31/131 (23.7%)	1	130/485 (26.8%)	0.055	42/151 (27.8%)	0.636	5162/23911 (21.6%)
Pain all over	59/1565 (3.8%)	4.80 × 10^–8^***	22/774 (2.8%)	0.196	2/131 (1.5%)	1	22/485 (4.5%)	4.80 × 10^–5^***	12/151 (7.9%)	0.004**	407/23911 (1.7%)
Sleep											
Insomnia	1263/1569 (80.5%)	0.006**	621/775 (80.1%)	0.210	104/131 (79.4%)	1	403/486 (82.9%)	0.011*	120/153 (78.4%)	1	18,421/23942 (76.4%)
Chronotype	554/1395 (39.7%)	0.575	283/695 (40.7%)	0.495	42/118 (35.6%)	1	179/433 (41.3%)	0.845	47/131 (35.9%)	1	7997/21289 (37.6%)
Sleep duration	594/1553 (38.2%)	2.63 × 10^–6^***	267/772 (34.6%)	0.789	46/129 (35.7%)	1	208/478 (43.5%)	7.50 × 10^–7^***	68/149 (45.6%)	0.003**	7639/23825 (32.1%)
Snoring	550/1423 (38.7%)	0.303	282/716 (39.4%)	0.413	44/113 (38.6%)	1	163/440 (37.0%)	1	54/136 (39.7%)	1	8029/22213 (36.1%)
Daytime sleepiness	72/1553 (4.7%)	4.61 × 10^–4^***	30/769 (3.9%)	0.570	3/126 (2.4%)	1	29/481 (6.0%)	4.03 × 10^–4^***	10/153 (6.5%)	0.065	683/23855 (2.9%)
Sleep pattern											
Health Sleep pattern	402/1249 (32.2%)	4.90 × 10^–4^***	208/642 (32.4%)	0.016*	34/99 (34.3%)	0.639	116/379 (30.6%)	0.011*	37/114 (32.5%)	0.350	7306/19674 (37.1%)
Intermediate sleep pattern	766/1249 (61.3%)	0.049*	397/642 (61.8%)	0.095	57/99 (57.6%)	0.939	237/379 (62.5%)	0.124	67/114 (58.8%)	1	11,502/19674 (58.5%)
Poor sleep pattern	81/1249 (6.5%)	7.67 × 10^–4^***	37/642 (5.8%)	0.121	8/99 (8.1%)	0.083	26/379 (6.9%)	0.030*	10/114 (8.8%)	0.042*	866/19674 (4.40%)
Sleep score	3(2–4)	9.16 × 10^–8^***	3(2–4)	4.37 × 10^–4^***	3(2–4)	0.517	3(2–4)	3.01 × 10^–5^***	3(2–4)	0.056	3(3–4)

Cognition	Median (range) [*n*]	*p* value	Median (range) [*n*]	*p* value	Median (range) [*n*]	*p* value	Median (range) [*n*]	*p* value	Median (range) [*n*]	*p* value	Median (range) [*n*]
Numeric memory	7 (6–7) [91]	1	7 (6–8) [29]	0.282	7 (6.5–7) [11]	1	6 (6–7) [30]	0.600	6 (5.5–7) [19]	0.524	7 (6–8) [2466]
Prospective memory	2 (1–2) [437]	0.287	2 (2–2) [218]	1	2 (1–2) [37]	1	2 (1–2) [128]	0.396	2 (1–2) [50]	0.077	2 (2–2) [8307]
Good recall	317/437 (72.5%)	0.090	168/218 (77.1%)	0.833	25/37 (67.6%)	0.300	90/128 (70.3%)	0.147	31/50 (62.0%)	0.029*	6331/8307 (76.2%)
Intermediate recall	93/437 (21.3%)	0.238	37/218 (17.0%)	0.531	12/37 (32.4%)	0.059	28/128 (21.9%)	0.458	15/50 (30.0%)	0.069	1569/8307 (18.9%)
Bad recall	27/437 (6.2%)	0.277	13/218 (6.0%)	0.577	0/37 (0.0%)	–	10/128 (7.8%)	0.193	4/50 (8.0%)	0.291	407/8307 (4.9%)
Fluid intelligence	6 (4–7) [413]	0.018*	6 (4–7.75) [206]	1	5 (4–5) [36]	0.306	6 (4–7) [123]	0.342	5 (4–7) [45]	0.268	6 (4–7) [8008]
Pairs matching											
Trial 1	0 (0–1) [1567]	0.128	0 (0–1) [775]	1	0 (0–1) [131]	1	0 (0–1) [485]	0.001**	0 (0–1) [152]	1	0 (0–1) [23795]
Trial 2	3 (2–6) [1567]	0.820	4 (2–6) [775]	0.640	3 (2–5) [131]	1	3 (2–6) [485]	1	4su (2–6) [152]	1	3 (2–6) [23795]

*p* values are all vs control and are represented as post-Bonferroni correction for multiple comparisons

*p < 0.05; **p < 0.01; ***p < 0.001

#### Sleep disturbance

Significantly higher rates of insomnia were present in dystonia (*p* = 0.006) and tremor groups (*p* = 0.011), as well as sub-optimal sleep duration (< 7 h or > 8 h) in the overall cohort (*p* = 2.63 × 10^–6^), dystonic tremor (*p* = 7.50 × 10^–7^) and unspecified forms of dystonia (*p* = 0.003) (Table 3). An excess of daytime sleepiness was also observed in the overall (*p* = 4.61 × 10^–4^) and tremor (*p* = 4.03 × 10^–4^) cohorts. The overall dystonia cohort (*p* = 9.16 × 10^–8^), cervical dystonia (*p* = 4.37 × 10^–4^) and tremor (*p* = 3.01 × 10^–5^) cohorts were also found to have significantly worse sleep scores compared to the control cohort, while poorer sleep patterns were described in the overall cohort (*p* = 7.67 × 10^–4^), dystonic tremor (*p* = 0.030) and unspecified forms of dystonia (*p* = 0.042). Consistent with this, there were fewer participants with reported healthy sleep patterns in the overall cohort (*p* = 4.90 × 10^–4^), cervical dystonia (*p* = 0.016) and dystonic tremor (*p* = 0.011) groups.

#### Cognition

Differences in cognitive scores, between dystonia and control cohorts, were less marked than that observed for the other non-motor symptoms. Here, overall lower fluid intelligence scores were observed (*p* = 0.018) (Table 3). Further examination found significantly fewer individuals in the unspecified dystonia cohort to have good prospective memory recall (*p* = 0.029). No significant differences were observed across the remaining cognitive assessment batteries.

#### Combined non-motor symptoms

**Fig. 1:**
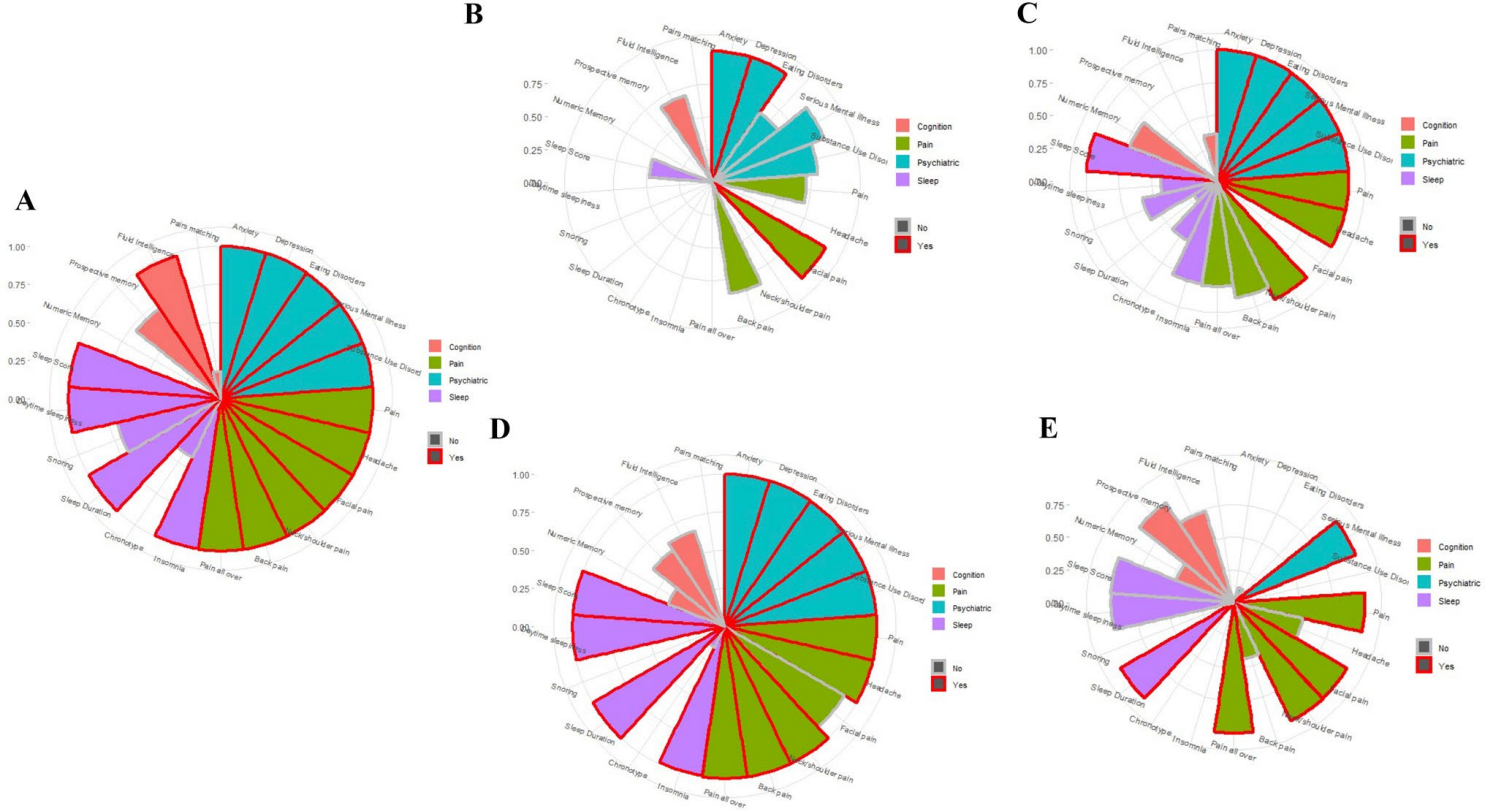
Coordinate plots for **a** dystonia overall, **b** blepharospasm, **c** cervical dystonia, **d** dystonic tremor and **e** other/unspecified dystonia. Bars represent 1- the Bonferroni adjusted *p* value with significant variables outlined in red. Bar colour fill represents the distinct non-motor symptom domains: pink (cognition), green (pain), blue (psychiatric) and purple (sleep)

A key advantage of the UKBB dataset is the opportunity to compare symptom rates and severity scores across multiple non-motor domains, as well as to compare the relative prevalence of these symptom clusters across multiple types of dystonia. These are visualised in Fig. 1, demonstrating prominent psychiatric and pain symptoms in the overall dystonia cohort, with a smaller cognitive profile and intermediate picture for sleep disturbance. However, this pattern clearly varies between dystonia subtypes with specific psychiatric and pain symptoms emerging in the blepharospasm group (Fig. 1B), with this differing from the more generalised excess of symptoms across these domains in the cervical (Fig. 1C) and dystonic tremor (Fig. 1D) groups. The dystonic tremor cohort also demonstrated the most prominent sleep disturbance profile, with significantly higher scores across four domains (insomnia, sleep duration, daytime sleepiness and poor sleep score), compared to single domains for the blepharospasm (sleep duration), cervical dystonia (poor sleep score) and unspecified forms of dystonia (sleep duration) (Fig. 1B, C, E). Visually, the relatively low impact of cognitive deficits is clearly observed, with only fluid intelligence deficits observed in the overall cohort.

### Mendelian causes of dystonia

**Fig. 2:**
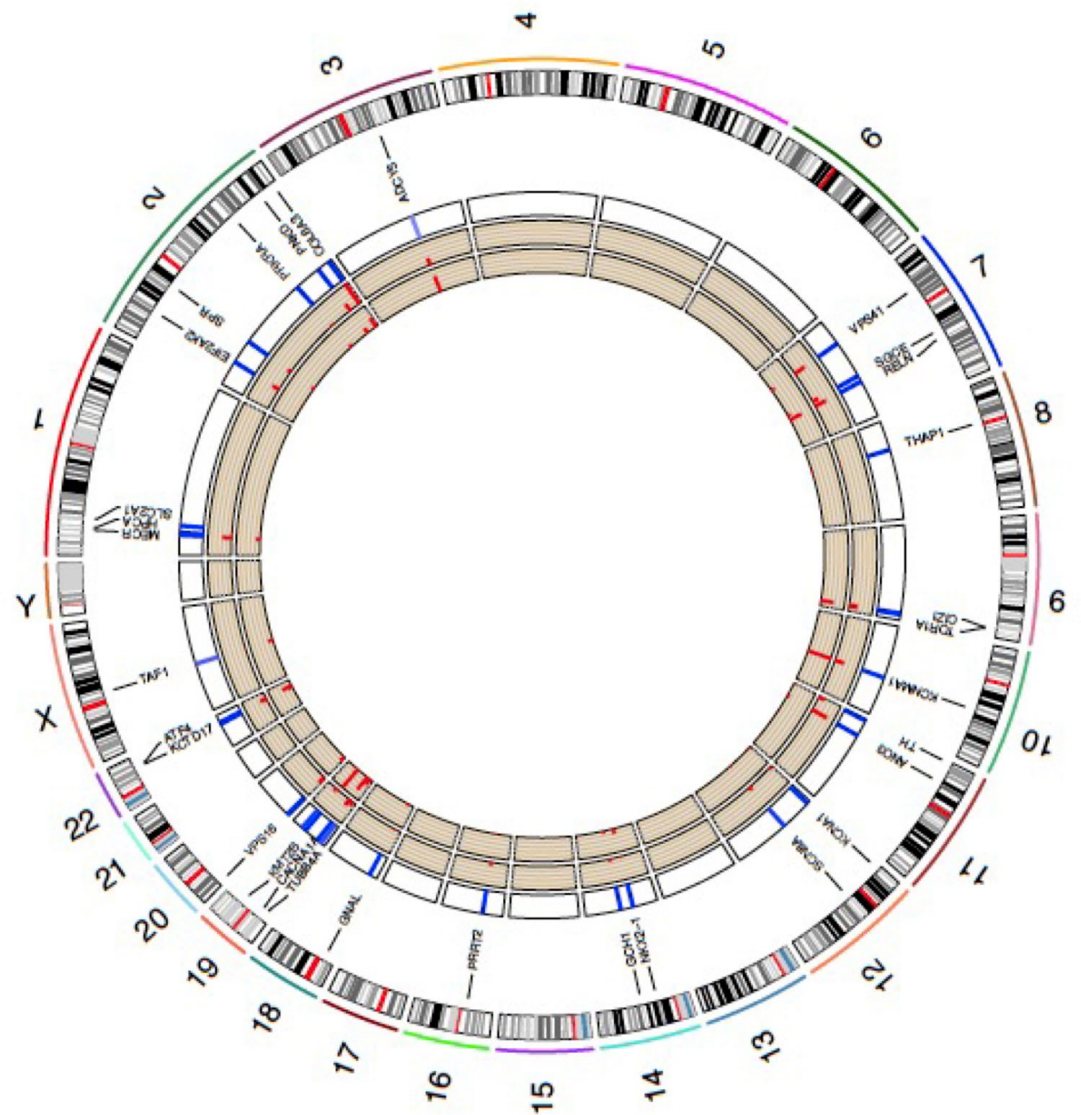
Circos plot of potentially pathogenic variants identified in Mendelian inherited genes with evidence of causation in dystonia. Outer tracks indicate individual chromosomes and the genomic location of genes linked with dystonia pathogenesis. Inner three tracks, moving from outermost to innermost, indicate the number of missense variants (MPC > 3) (heatmap), loss of function variants (histogram) and other forms of non-synonymous variants (histogram)

Exome sequencing data were available for 200,643 individuals within UKBB. Following assessment of relatedness and ancestry 21,601 individuals were removed from onward analysis, including 48 individuals identified within the dystonia cohort, resulting in an overall cohort of *n* = 179,028, of which 485 were within the dystonic cohort. Loss of function (*n* = 831) and other types of non-synonymous variants (including inframe deletion, inframe insertion, stop loss and start loss variants, *n* = 331) were identified in all of the genes investigated (Table 4), while 83 missense variants with an MPC score > 3 were observed in the *ADCY5*, *KCNMA1*, *KCNA1*, *SCN8A*, *GNAL*, *CACNA1A*, *TUBB4A*, *ATP1A3* and *TAF1* genes (Fig. 2). Eighty-five of these variants have been reported previously with 39 described as pathogenic/likely pathogenic (*SCN8A*, *SPR*, *COL6A3*, *ADCY5*, *SGCE*, *RELN*, *TOR1A*, *THAP1*, *KCNMA1*, *TH*, *GCH1*, *PRRT2*, *VPS16* and *CACNA1A* genes), 38 of uncertain significance (*ADCY5*, *SCN8A*, *TUBB4A*, *ATP1A3*, *PNKD*, *COL6A3*, *RELN*, *TOR1A*, *CIZ1*, *GNAL*, *KCNA1*, *ANO3*, *KCNMA1* and *CACNA1A* genes) and 8 benign/likely benign (*CIZ1*, *SCN8A*, *KCNMA1*, *CACNA1A* genes). Nine of those previously reported as pathogenic/likely pathogenic were linked with neurological or mental health diagnoses within the UKBB dataset, all being loss of function variants (Supplementary Table 4). These included *COL6A3* (*n* = 3), previously described in DYT27 and Bethlem Myopathy cohorts, but linked with sleep apnoea and carpal tunnel phenotypes here. Those involving *THAP1* (*n* = 1) and *TOR1A* (*n* = 1) were linked with myelopathies, while *SGCE* variants (*n* = 2) were associated with depression, anxiety and myoclonus, and hemiplegia for the single overlapping *TH* variant. The single variant (loss of function) identified within the dystonia cohort (*ANO3*) was linked with a clinical diagnosis of cervical dystonia and has not been previously reported (Supplementary Table 4, highlighted in bold).

**Table 4:** Number of individuals from the complete genetic cohort (*n* = 179,028) and dystonia cohort (*n* = 485) with genetic variants in 32 genes that have previously been associated with dystonia

Gene	Location	OMIM number	Synonymous variants		Missense variants						Loss of function variants		Other non-synonymous variants	
					MPC < 2		MPC 2–3		MPC > 3					
			*S*	*R*	*S*	*R*	*S*	*R*	*S*	*R*	*S*	*R*	*S*	*R*
*SLC2A1*	1p34.2	138140	36; 0	114; 1	31; 0	82; 0	18; 0	34; 0	0; 0	0; 0	2; 0	0; 0	0; 0	0; 0
*HPCA*	1p35.1	142622	12; 0	43; 0	19; 1	42; 0	9; 0	5; 0	0; 0	0; 0	1; 0	3; 0	1; 0	0; 0
*MECR*	1p35.3	608205	20; 0	61; 0	64; 0	152; 1	0; 0	0; 0	0; 0	0; 0	14; 0	27; 0	4; 0	3; 0
*SPR*	2p13.2	182125	21; 0	36; 0	44; 0	77; 0	0; 0	0; 0	0; 0	0; 0	4; 0	12; 0	2; 0	2; 0
*EIF2AK2*	2p22.2	176871	25; 0	60; 0	68; 0	135; 0	0; 0	0; 0	0; 0	0; 0	14; 0	18; 0	1; 0	0; 0
*PRKRA*	2q31.2	603424	16; 0	25; 0	22; 0	58; 1	2; 0	0; 0	0; 0	0; 0	4; 0	5; 0	3; 0	2; 0
*PNKD*	2q35	609023	34; 0	71; 0	58; 0	166; 0	0; 0	0; 0	0; 0	0; 0	12; 0	21; 0	1; 0	9; 0
*COL6A3*	2q37.3	120250	189; 0	553; 2	484; 0	1222; 8	0; 0	0; 0	0; 0	0; 0	35; 0	65; 0	4; 0	14; 0
*ADCY5*	3q21.1	600293	93; 0	280; 1	153; 0	291; 2	40; 0	58; 0	3; 0	22; 0	17; 0	14; 0	6; 0	23; 0
*VPS41*	7p14.1	605485	44; 0	90; 1	110; 0	212; 3	0; 0	0; 0	0; 0	0; 0	20; 0	30; 0	2; 0	4; 0
*SGCE*	7q21.3	604149	25; 1	42; 0	62; 0	137; 0	0; 0	0; 0	0; 0	0; 0	13; 0	16; 0	2; 0	3; 0
*RELN*	7q22.1	600514	195; 0	429; 0	507; 1	882; 2	6; 0	9; 0	0; 0	0; 0	31; 0	20; 0	8; 0	14; 0
*THAP1*	8p11.21	609520	10; 0	13; 0	21; 0	61; 0	0; 0	0; 0	0; 0	0; 0	3; 0	2; 0	2; 0	0; 0
*TOR1A*	9q34.11	605204	27; 0	46; 0	51; 0	111; 1	0; 0	0; 0	0; 0	0; 0	8; 0	28; 0	2; 0	2; 0
*CIZ1*	9q34.11	611420	56; 1	124; 0	107; 0	233; 0	0; 0	0; 0	0; 0	0; 0	24; 0	12; 0	5; 0	17; 0
*KCNMA1*	10q22.3	600150	95; 0	243; 0	66; 0	171; 0	49; 0	50; 0	3; 0	0; 0	22; 0	19; 0	9; 0	33; 0
*ANO3*	11p14.3	610110	71; 0	122; 0	115; 0	221; 1	19; 1	25; 0	0; 0	0; 0	25; 1	45; 0	2; 0	2; 0
*TH*	11p15.5	191290	35; 0	91; 1	92; 1	252; 0	2; 0	0; 0	0; 0	0; 0	10; 0	19; 0	2; 0	0; 0
*KCNA1*	12p13.32	176260	35; 1	78; 0	25; 0	46; 0	30; 0	25; 0	0; 0	4; 0	7; 0	3; 0	2; 0	0; 0
*SCN8A*	12q13.13	600702	136; 1	236; 1	86; 1	230; 2	65; 0	124; 1	6; 0	2; 0	7; 0	10; 0	2; 0	0; 0
*NKX2-1*	14q13.3	600635	34; 0	67; 0	0; 0	0; 0	0; 0	0; 0	0; 0	0; 0	1; 0	6; 0	0; 0	9; 0
*GCH1*	14q22.2	600225	15; 0	65; 0	21; 0	54; 0	11; 0	9; 0	0; 0	0; 0	4; 0	7; 0	1; 0	3; 0
*PRRT2*	16p11.2	614386	19; 0	77; 0	79; 1	155; 1	0; 0	0; 0	0; 0	0; 0	6; 0	11; 0	2; 0	0; 0
*GNAL*	18p11.21	139312	24; 0	65; 0	27; 0	37; 1	25; 0	41; 0	3; 0	0; 0	5; 0	2; 0	3; 0	0; 0
*CACNA1A*	19p13.13	601011	180; 0	439; 1	251; 0	594; 3	109; 1	179; 0	2; 0	0; 0	18; 0	12; 0	4; 0	24; 0
*TUBB4A*	19p13.3	602662	28; 0	129; 0	23; 0	56; 0	24; 1	18; 1	8; 0	5; 0	12; 0	9; 0	2; 0	10; 0
*KMT2B*	19q13.12	606834	235; 0	584; 2	0; 0	0; 0	0; 0	0; 0	0; 0	0; 0	6; 0	0; 0	10; 0	30; 0
*ATP1A3*	19q13.2	182350	95; 0	209; 1	32; 0	71; 1	47; 0	44; 0	5; 0	4; 0	9; 0	6; 0	1; 0	0; 0
*VPS16*	20p13	608550	56; 0	108; 1	99; 1	348; 1	0; 0	0; 0	0; 0	0; 0	15; 0	17; 0	0; 0	10; 0
*KCTD17*	22q12.3	616386	9; 0	41; 0	27; 0	58; 1	7; 0	27; 0	0; 0	0; 0	7; 0	3; 0	6; 0	2; 0
*ATF4*	22q13.1	604064	47; 0	97; 1	81; 0	210; 1	0; 0	0; 0	0; 0	0; 0	10; 0	19; 0	3; 0	15; 0
*TAF1*	Xq13.1	313650	72; 0	124; 0	95; 0	163; 1	34; 0	41; 0	7; 0	9; 0	1; 0	2; 0	4; 0	4; 0

Number of variants identified per gene. Results given as overall whole exome sequencing (WES) dataset (*n* = 200,643); dystonia cohort (*n* = 638) with each set of values presented as whole cohort (inclusive of dystonia cohort); dystonia cohort only. MPC: missense badness, PolyPhen-2 and constraint pathogenicity classifier. R: rare variant (present 2–5 times in the dataset), S: singleton variants (present once in the dataset). Other non-synonymous variants include inframe deletion, inframe insertion, stop loss and start loss variants

### BMPMM analysis

**Fig. 3:**
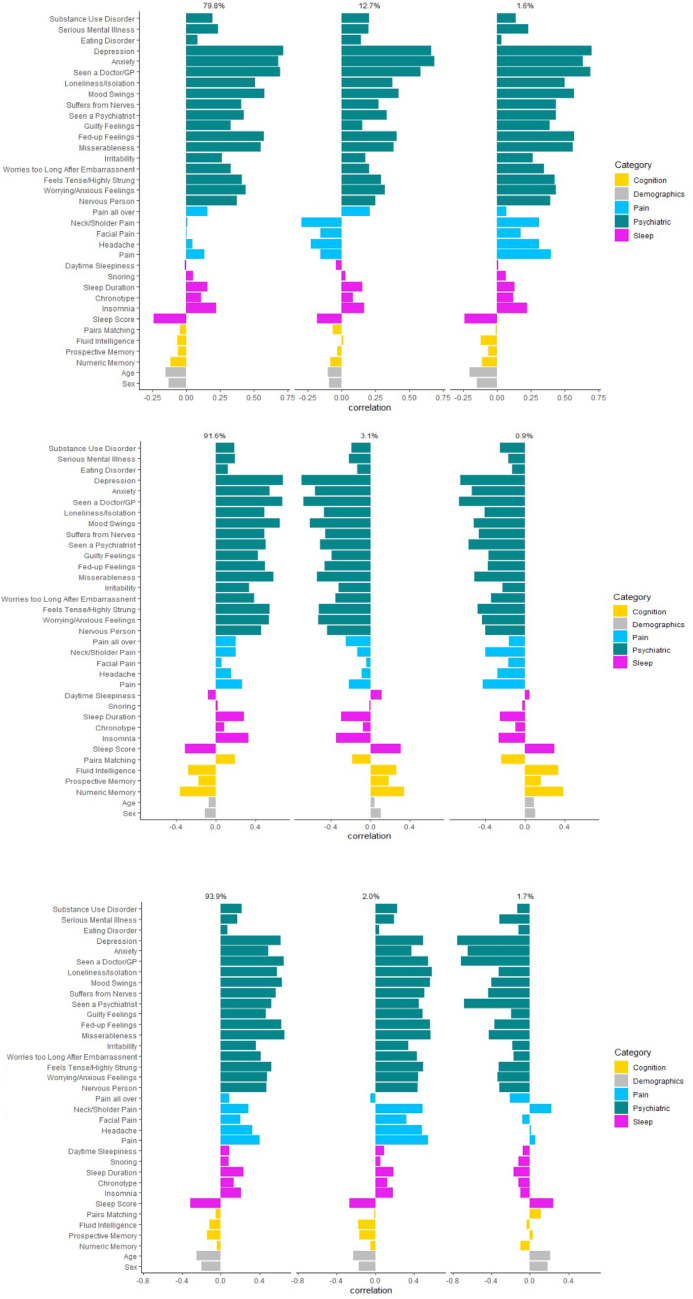
Three genetically informed phenotypic axes that represented the most phenotypic variance for **A** the overall dystonia cohort, **B** cervical dystonia and **C** dystonic tremor. Above each axis is the proportion of phenotypic variation it represents. Seen a GP/doctor and seen psychiatrist refer to seeing the specified professional for nerves, anxiety, tension, or depression. A positive correlation (bars going to the right) indicates increased psychiatric, pain and sleep disturbance, but improved cognitive performance

Having excluded the single dystonia case harbouring a non-synonymous variant on WES analysis, SNP genotype data used in this analysis were available for 1387/1572 (88.2%) of the overall dystonia cohort, with cervical dystonia and dystonic tremor cohorts also met our criteria of a minimum of 400 participants with both genotype and phenotype data. Using this approach, multiple axes were generated within each clinical cohort; however, we have chosen to focus on the three axes with the highest degree of variation and therefore likelihood of clinical relevance (Fig. 3). Key to this analysis is individual bar size, indicating the relative contribution of that symptom/symptom group to the overall phenotype. With this in mind, evident across the overall cohort and individual diagnoses (cervical dystonia and dystonic tremor) psychiatric symptoms formed the predominant non-motor symptom. In addition, each motor clinical group had a single predominant non-motor axis, 79.8%, 91.6% and 93.9% for the overall, cervical dystonia and tremor cohorts, respectively. Within the overall cohort, the main axis (79.8%) demonstrated a predominantly psychiatric phenotype, with sleep disturbance next most evident, but with only ~ 1/3rd of the effect of the psychiatric symptoms. The second and third axes (12.7% and 1.6%) differ predominantly in the relative contribution of pain symptoms, with this most evident in axis three (1.6% of clinical variance). Amongst those diagnosed with cervical dystonia (*n* = 694), the single predominant axis (91.6%) demonstrated a predominant psychiatric phenotype, mainly involving symptoms of anxiety and depression. Pain and sleep disturbance were also contributory symptoms, although only approximately half that of the contribution of psychiatric symptoms (Fig. 3B). The two remaining axes (3.1% and 0.9% non-motor variance) suggested more prominent cognitive involvement, with difficulties associated with fluid intelligence, prospective and numeric memory. Psychiatric, pain and sleep disturbance formed the predominant non-motor symptoms in the two main axes for the dystonia with tremor cohort (*n* = 442) (93.9% and 2.0% of clinical variance), while the third axis (1.7%) suggested an overall minimal non-motor phenotype.

## Discussion

This study makes use of the broad clinical phenotypic and genetic data available within the UKBB to systematically examine multiple non-motor symptoms within the same dystonia cohort, including distinct dystonia subtypes and represents the largest of its kind in the dystonia field to date. Key findings include: (a) an excess of non-motor symptoms in those with dystonia compared to controls, predominantly involving psychiatric symptoms, pain and disturbed sleep, with variation dependent on dystonia subtype, (b) low rate of potentially pathogenic variants in known dystonia causing genes within the identified cohort (1 ANO3 variant in 485 individuals diagnosed with dystonia), within the overall cohort (*n* = 179,028) 1,245 non-synonymous variants were identified, 85 reported previously and 39 of these considered pathogenic, (c) combining genetic and non-motor phenotypic data identified multiple axes of phenotypic variation within the dystonia cohorts, with psychiatric symptoms again forming the predominant non-motor symptom, with smaller effects of pain and sleep disturbance, suggesting potential for varying biological mechanisms underpinning the phenotypic heterogeneity observed in clinical practice.

Combining data gathered from primary care and hospital admission records, as well as patient reported symptoms, this study demonstrates psychiatric symptomatology to be the most pronounced non-motor symptom in dystonia. These findings are not only consistent with the results of work published to date, but also support the emphasis of evaluation of psychiatric symptoms over other non-motor components in both Mendelian and idiopathic dystonia cohort studies to date [4, 6, 7, 39]. This study also reinforces depression and anxiety being the predominant psychiatric symptoms observed, while also identifying other diagnoses, such as eating disorders, largely under explored to date but warranting further investigation [40]. However, the pattern and predominance of these psychiatric symptoms is also shown to vary between dystonia subtypes; for example, depression and serious mental illness were predominant in blepharospasm and unspecified forms of dystonia, while a broader groups of symptoms including anxiety disorder and substance abuse were observed in cervical dystonia and dystonic tremor (Fig. 1) [41, 42].

Of the other non-motor symptom groups explored, pain and sleep disturbance were also consistently reported, although their impact appears to be approximately half that of psychiatric disturbance. Pain has long been recognised as a prominent feature in cervical dystonia and, as observed in this study, tending to focus on the motor affected region [43]. Pain is less well reported in blepharospasm, but was again centred on the motor affected region, potentially indicating that some element of this may be a secondary phenomenon. Within the overall dystonia cohort, specific areas of sleep disturbance included insomnia, reduced sleep duration and daytime sleepiness, in part consistent with previous work [9]. However, the sleep questionnaires used during UKBB assessment differ from the standardised, sleep specific questionnaires typically used in previous studies of dystonia and lack the depth of understanding provided by investigations such as polysomnography [44].

A key finding from this study was the remarkably small contribution of cognitive disturbance to the non-motor symptom spectrum observed across the overall dystonia cohort, and in particular cervical dystonia and dystonic tremor subtypes, in the context of robust cognitive assessment. Fluid intelligence, in the overall cohort, and prospective memory in the blepharospasm and undefined subgroups were the only domains significantly impaired compared to controls. Previous cross-sectional studies, however, have demonstrated impairment to a wider group of cognitive domains, including impairments to working memory, processing speed and short-term memory [45]. These differences in study outcomes may in part relate to the few and subtle cognitive deficits that likely exist in dystonia; however, it should also be noted that completion rates of the cognitive assessments within this cohort were the lowest across the non-motor symptoms examined and may have contributed to the outcomes observed [46]. Interestingly, however, other studies do support the more specific deficit in prospective memory observed here, with some indicating a selective deficit of time-based dysfunction in this domain [16].

Due to the nature of recruitment to the UKBB and its resultant demographic characteristics, this study doesn’t represent a true population-level analysis. However, the genetic analysis represents one of the largest cohorts in which Mendelian inherited genes-associated disease causation of dystonia has been examined. A total of 1,245 potentially pathogenic non-synonymous variants in the 32 dystonia genes were identified, including 831 loss of function, 83 missense (MPC score > 3) and 331 other non-synonymous forms. Eighty-five of these have been reported previously with 45.9% (39/85) considered pathogenic/likely pathogenic. With the exception of one, all were reported within the non-dystonia cohort, suggesting known contribution of incomplete penetrance in some known dystonia causing genes, such as *THAP1* (DYT6) and *TOR1A* (DYT1). Within the dystonia cohort, only a single *ANO3* loss of function variant was observed, associated with a documented cervical dystonia phenotype, but not previously reported. To date, missense mutations are the most commonly reported mutation type in relation to ANO3-dystonia pathology, with no previously reported loss of function mutations in this context. The findings within this cohort are therefore of uncertain significance, and future work is needed to determine whether mutations of this type impact protein expression and function. By comparison, the only previous study using next-generation genetic sequencing of dystonia genes at this scale examined 764 individuals with a clinical diagnosis of dystonia, identifying causative or likely causative variants in 135/728 (19%) families, involving 78 distinct monogenic disorders [47]. However, half of this cohort were described as having developmental delay or hypotonia, and approximately a third with intellectual disability or speech disorder. Individuals with these levels of co-morbidity are unlikely to have been recruited to the UKBB, and therefore, these differing rates of variants likely reflect the overall clinical context of the cohort.

Despite clinical phenotypic heterogeneity being widely recognised across multiple neurological disorders, notably adult-onset idiopathic forms in relation to dystonia, bioinformatic tools enabling combined phenotypic and genotypic analyses have emerged only relatively recently [48]. Here we quantified diverse patient phenotypes on a continuous scale via the use of phenotype axes. This approach overcomes many of the limitations associated with the clustering methods previously used to classify dystonia heterogeneity. The phenotypic axes are extremely robust in term of clinical features considered and enable potential alignment of multiple cohorts with different clinical structure. These universal axes have the potential to accelerate our understanding of how dystonia presents in individual patients, providing more robust and objective quantitative traits through which patients may appropriately be grouped and compared. The most pronounced genetically influenced phenotypic axes identified in the overall dystonic cohort in this study were in keeping with our previous work, in independent cohorts, and using only clinical phenotypes. This study involved a substantially larger cohort, as well as being combined with SNP genetic data, indicating both a consistency to these phenotypic findings and the likelihood of a polygenic contribution to the observed clinical heterogeneity [49]. The most pronounced genetically influenced phenotypic axes were also in keeping with the findings from the direct case–control analysis for both overall and individual dystonia diagnostic groups, again supporting a potential genetic contribution to this phenotype [5, 6, 50]. The most prominent axis across all three groups analysed (overall cohort, cervical dystonia and dystonic tremor) was that of mood related psychiatric symptoms (depression and anxiety), coupled with pain and disturbed sleep, a pattern of symptoms beginning to emerge consistently across multiple studies [11]. Although Genome-Wide Association Studies (GWAS) have been undertaken in dystonia, in comparison with other disease groups these have involved relatively small cohorts. Larger, multi-centre studies of this kind will be needed to better understand which genes, and their size effects, are potentially contributing to this polygenic component [51].

Whilst this study benefits from the depth of clinical data available within the UKBB, as well as the opportunity to examine multiple dystonia types using an identical battery of assessments, several limitations need to be considered. The most prominent is the lack of clinical confirmation of diagnosis, not possible in an anonymised cohort and requiring substantial time and resource for a cohort of this size. However, in deriving this cohort we have used a previously published pipeline in which a proportion of the cohort (*n* = 90) underwent clinical confirmation of their dystonia diagnosis through in person examination [20]. In addition, only ~ 43% of English GP data is linked with the UKBB, making the cohort identified here a likely underestimation of all those diagnosed with dystonia within the UKBB. Additionally, we have applied a very stringent clinical code exclusion criteria with the aim of minimising the potential for inclusion of cases of secondary dystonia within the cohort. However, in so doing we have likely omitted cases of primary dystonia when present with another movement disorder (e.g. myoclonus) providing a further potential source of cohort size underestimation. A minimum of 100 cases was also applied to ongoing analysis of the individual dystonia subtypes, with only cervical dystonia, blepharospasm, dystonic tremor and unspecified forms of dystonia meeting this threshold. This approach resulted in other subtypes, for example, writer’s cramp and orofacial dystonia, being excluded from onward analysis and therefore loss of opportunity for better understanding of their non-motor symptom profile. Future work will require investigation of larger cohorts with this allowing for further dystonia subtypes to be included in more in depth analysis. Finally, the dystonia and control cohorts were not matched for educational attainment, which has been shown to impact pain recognition in dystonia which may have influenced some of the differences observed. However, previous work has shown UKBB participants overall to have a higher level of educational attainment, potentially further impacting the outcomes of the pain assessments reported here [52].

In summary, this study represents one of the largest dystonia cohorts in whom in depth non-motor symptom assessment has been undertaken to date, as well as enabling direct comparison between distinct forms of dystonia. We have shown an excess of non-motor symptoms across all dystonia groups, with this demonstrating a predominant psychiatric phenotype coupled with smaller but sizeable components of pain and sleep impairment. This work demonstrates the need for integration of non-motor symptom evaluation during routine clinical practice, as is observed with multiple other neurological disorders, and for these to be evaluated at regular intervals to allow for identification of non-motor symptom variation as well as the implementation of appropriate treatment or therapy where needed. Next-generation sequencing analysis has identified novel variants with potential for pathogenicity and needing further analysis, while integration of genetic SNP data demonstrated phenotypic variability within each cohort, mirroring the symptomatic heterogeneity observed in clinical practice and lending support that these traits contribute to the primary phenotype of dystonia.

## Supplementary Information

Below is the link to the electronic supplementary material.

Supplementary file1 (DOCX 15 KB)

Supplementary file2 (DOCX 22 KB)

Supplementary file3 (DOCX 32 KB)

Supplementary file4 (XLSX 78 KB)

## References

[1] Girach A, Vinagre Aragon A, Zis P (2019). Quality of life in idiopathic dystonia: a systematic review. J Neurol.

[2] Albanese A, Bhatia K, Bressman SB, Delong MR, Fahn S, Fung VSC (2013). Phenomenology and classification of dystonia: a consensus update. Mov Disord.

[3] Moraru E, Schnider P, Wimmer A, Wenzel T, Birner P, Griengl H (2002). Relation between depression and anxiety in dystonic patients: implications for clinical management. Depress Anxiety.

[4] Peall KJ, Smith DJ, Kurian MA, Wardle M, Waite AJ, Hedderly T (2013). SGCE mutations cause psychiatric disorders: clinical and genetic characterization. Brain.

[5] Ferrazzano G, Berardelli I, Conte A, Baione V, Concolato C, Belvisi D (2019). Motor and non-motor symptoms in blepharospasm: clinical and pathophysiological implications. J Neurol.

[6] Novaretti N, Cunha ALN, Bezerra TC, Pereira MAP, De Oliveira DS, Macruz Brito MMC (2019). The prevalence and correlation of non-motor symptoms in adult patients with idiopathic focal or segmental dystonia. Tremor Other Hyperkinet Mov.

[7] Müller J, Kemmler G, Wissel J, Schneider A, Voller B, Grossmann J (2002). The impact of blepharospasm and cervical dystonia on health-related quality of life and depression. J Neurol.

[8] Charles PD, Adler CH, Stacy M, Comella C, Jankovic J, Manack Adams A (2014). Cervical dystonia and pain: characteristics and treatment patterns from CD PROBE (Cervical Dystonia Patient Registry for Observation of OnabotulinumtoxinA Efficacy). J Neurol.

[9] Hertenstein E, Tang NKY, Bernstein CJ, Nissen C, Underwood MR, Sandhu HK (2015). Sleep in patients with primary dystonia: a systematic review on the state of research and perspectives. Sleep Med Rev.

[10] Sun Y, Tsai P-J, Chu C-L, Huang W-C, Bee Y-S (2018). Epidemiology of benign essential blepharospasm: a nationwide population-based retrospective study in Taiwan. PLoS ONE.

[11] Smit M, Kamphuis ASJ, Bartels AL, Han V, Stewart RE, Zijdewind I (2017). Fatigue, sleep disturbances, and their influence on quality of life in cervical dystonia patients. Mov Disord Clin Pract.

[12] Antelmi E, Ferri R, Provini F, Scaglione CML, Mignani F, Rundo F (2017). Modulation of the muscle activity during sleep in cervical dystonia. Sleep.

[13] Feuerstein J, Holden S, Sillau S, Berman B (2020) Social cognition is abnormal in cervical dystonia but not blepharospasm (5224). Neurology 94 10.1002/mdc3.13808 PMC10450232 37635782

[14] Monaghan R, Cogley C, Burke T, McCormack D, O’Riordan S, Ndukwe I (2021). Non-motor features of cervical dystonia: cognition, social cognition, psychological distress and quality of life. Clin Park Relat Disord.

[15] Czekóová K, Zemánková P, Shaw DJ, Bareš M (2017). Social cognition and idiopathic isolated cervical dystonia. J Neural Transm.

[16] Maggi G, D’Iorio A, Mautone G, Peluso S, Manganelli F, Dubbioso R (2019). Cognitive correlates of prospective memory in dystonia. Park Relat Disord.

[17] Dias FMV, Doyle FCP, Kummer A, Cardoso F, Caramelli P, Teixeira AL (2009). Executive functioning in patients with blepharospasm in comparison with patients with hemifacial spasm. Arq Neuropsiquiatr.

[18] Alemán GG, De Erausquin GA, Micheli F (2009). Cognitive disturbances in primary blepharospasm. Mov Disord.

[19] Jacobs BM, Belete D, Bestwick J, Blauwendraat C, Bandres-Ciga S, Heilbron K (2020). Parkinson’s disease determinants, prediction and gene-environment interactions in the UK Biobank. J Neurol Neurosurg Psychiatry.

[20] Bailey GA, Rawlings A, Torabi F, Pickrell O, Peall KJ (2022). Adult-onset idiopathic dystonia: a national data-linkage study to determine epidemiological, social deprivation, and mortality characteristics. Eur J Neurol.

[21] Bycroft C, Freeman C, Petkova D, Band G, Elliott LT, Sharp K (2018). The UK Biobank resource with deep phenotyping and genomic data. Nature.

[22] Elliott P, Peakman TC (2008). The UK Biobank sample handling and storage protocol for the collection, processing and archiving of human blood and urine. Int J Epidemiol.

[23] Glanville KP, Coleman JRI, Howard DM, Pain O, Hanscombe KB, Jermy B (2021). Multiple measures of depression to enhance validity of major depressive disorder in the UK Biobank. BJPsych Open.

[24] Carvalho-e-Silva AP, Harmer AR, Ferreira ML, Ferreira PH (2021). The effect of the anti-diabetic drug metformin on musculoskeletal pain: a cross-sectional study with 21,889 individuals from the UK biobank. Eur J Pain (United Kingdom).

[25] Meng W, Chan BW, Harris C, Freidin MB, Hebert HL, Adams MJ (2020). A genome-wide association study finds genetic variants associated with neck or shoulder pain in UK Biobank. Hum Mol Genet.

[26] Fan M, Sun D, Zhou T, Heianza Y, Lv J, Li L (2020). Sleep patterns, genetic susceptibility, and incident cardiovascular disease: a prospective study of 385,292 UK biobank participants. Eur Heart J.

[27] Kyle SD, Sexton CE, Feige B, Luik AI, Lane J, Saxena R (2017). Sleep and cognitive performance: cross-sectional associations in the UK Biobank. Sleep Med.

[28] Fawns-Ritchie C, Deary IJ (2020). Reliability and validity of the UK Biobank cognitive tests. PLoS ONE.

[29] Van Hout CV, Tachmazidou I, Backman JD, Hoffman JD, Liu D, Pandey AK (2020). Exome sequencing and characterization of 49,960 individuals in the UK Biobank. Nature.

[30] The Hail Team (2008) Hail. https://github.com/hail-is/hail

[31] McLaren W, Gil L, Hunt SE, Riat HS, Ritchie GRS, Thormann A (2016). The ensembl variant effect predictor. Genome Biol.

[32] Mencacci NE, Reynolds R, Ruiz SG, Vandrovcova J, Forabosco P, Sánchez-Ferrer A (2020). Dystonia genes functionally converge in specific neurons and share neurobiology with psychiatric disorders. Brain.

[33] Samocha KE, Kosmicki JA, Karczewski KJ, O’Donnell-Luria AH, Pierce-Hoffman E, MacArthur DG (2017). Regional missense constraint improves variant deleteriousness prediction. BioRxiv.

[34] Zhou X, Stephens M (2012). Genome-wide efficient mixed-model analysis for association studies. Nat Genet.

[35] Zhou X, Carbonetto P, Stephens M (2013). Polygenic modeling with Bayesian sparse linear mixed models. PLoS Genet.

[36] Zhou X, Stephens M (2014). Efficient multivariate linear mixed model algorithms for genome-wide association studies. Nat Methods.

[37] Dahl A, Iotchkova V, Baud A, Johansson S, Gyllensten U, Soranzo N (2016). A multiple-phenotype imputation method for genetic studies. Nat Genet.

[38] Okosieme OE, Taylor PN, Evans C, Thayer D, Chai A, Khan I (2019). Primary therapy of Graves’ disease and cardiovascular morbidity and mortality: a linked-record cohort study. Lancet Diabetes Endocrinol.

[39] Berman BD, Junker J, Shelton E, Sillau SH, Jinnah HA, Perlmutter JS (2017). Psychiatric associations of adult-onset focal dystonia phenotypes. J Neurol Neurosurg Psychiatry.

[40] Ellement B, Jasaui Y, Kathol K, Nosratmirshekarlou E, Pringsheim T, Sarna J (2020). Social cognition in cervical dystonia: phenotype and relationship to anxiety and depression. Eur J Neurol.

[41] Yang J, Shao N, Song W, Wei Q, Ou R, Wu Y (2017). Nonmotor symptoms in primary adult-onset cervical dystonia and blepharospasm. Brain Behav.

[42] Mahajan A, Jankovic J, Marsh L, Patel A, Jinnah HA, Comella C (2018). Cervical dystonia and substance abuse. J Neurol.

[43] Tinazzi M, Squintani GM, Bhatia KP, Segatti A, Donato F, Valeriani M (2019). Pain in cervical dystonia: evidence of abnormal inhibitory control. Park Relat Disord.

[44] Ray S, Kumar G, Kutty B, Pramod Pal K, Yadav R (2021). Patients with primary cervical dystonia have significant sleep impairment and polysomnographic abnormalities. Sleep Vigil.

[45] Romano R, Bertolino A, Gigante A, Martino D, Livrea P, Defazio G (2014). Impaired cognitive functions in adult-onset primary cranial cervical dystonia. Park Relat Disord.

[46] Jahanshahi M, Torkamani M (2017). The cognitive features of idiopathic and DYT1 dystonia. Mov Disord.

[47] Zech M, Jech R, Boesch S, Škorvánek M, Weber S, Wagner M (2020). Monogenic variants in dystonia: an exome-wide sequencing study. Lancet Neurol.

[48] Pengelly RJ, Alom T, Zhang Z, Hunt D, Ennis S, Collins A (2017). Evaluating phenotype-driven approaches for genetic diagnoses from exomes in a clinical setting. Sci Rep.

[49] Wadon ME, Bailey GA, Yilmaz Z, Hubbard E, AlSaeed M, Robinson A (2021). Non-motor phenotypic subgroups in adult-onset idiopathic, isolated, focal cervical dystonia. Brain Behav.

[50] Smit M, Kuiper A, Han V, Jiawan VCR, Douma G, van Harten B (2016). Psychiatric co-morbidity is highly prevalent in idiopathic cervical dystonia and significantly influences health-related quality of life: results of a controlled study. Park Relat Disord.

[51] Sun YV, Li C, Hui Q, Huang Y, Barbano R, Rodriguez R (2021). A multi-center genome-wide association study of cervical dystonia. Mov Disord.

[52] Fry A, Littlejohns TJ, Sudlow C (2017). Comparison of sociodemographic and health-related characteristics if UK Biobank participants with those of the general population. Am J Epidemiol.

