# Supplementary material for: Clinical and genotypic analysis in determining dystonia non-motor phenotypic heterogeneity: a UK Biobank study
Source: J Neurol. 2022 Aug 4;269(12):6436–51. doi: 10.1007/s00415-022-11307-4 (PMC9618530; doi:10.1007/s00415-022-11307-4)
Supplement: Supplementary file 1 — Supplementary file1 (DOCX 15 KB) [file 415_2022_11307_MOESM1_ESM.docx]

**Supplementary Table 1: Inclusion codes for UK Biobank dystonia cohort**

|  | | | | | |
| --- | --- | --- | --- | --- | --- |
| **Dystonia subtype** | **ICD-10 Code** | **Read Code** | | | **Read Code Description** |
| Idiopathic torsion dystonia |  |  | F136. |  | Idiopathic torsion dystonia |
|  |  |  | F137. |  | Symptomatic torsion dystonia |
|  |  |  | F137y |  | Symptomatic torsion dystonia OS |
|  |  |  | F137z |  | Symptomatic torsion dystonia NOS |
|  |  |  | F138. |  | Fragment of torsion dystonia |
|  |  |  | F138z |  | Torsion dystonia fragment NOS |
| Idiopathic nonfamilial dystonia | G24.2 |  |  |  |  |
| Idiopathic familial dystonia | G24.1 |  | F1360 |  | Idiopathic familial dystonia |
| Cervical dystonia | G24.3 |  | F1382 |  | Spasmodic torticollis |
|  |  |  | 16A3. |  | Torticollis - symptom |
|  |  |  | N135. |  | Torticollis unspecified |
|  |  |  | N1350 |  | Intermittent torticollis |
|  |  |  | N135z |  | Torticollis NOS |
| Idiopathic Orofacial dystonia | G24.4 |  |  |  |  |
| Blepharospasm | G24.5 |  | F1380 |  | Blepharospasm |
| Writer’s cramp |  |  | F1383 |  | Organic Writer’s cramp |
| Myoclonic dystonia |  |  | F13B. |  | Myoclonic dystonia |
| Segawa syndrome |  |  | F13C. |  | Segawa syndrome |
| Other | G24.8 |  | Fyu24 |  | [X]Other dystonia |
| Unspecified | G24.9 |  | Fyu2A |  | [X]Dystonia, unspecified |
|  |  |  | F13X. |  | Dystonia, unspecified |
| Tremor |  |  | 1B22. |  | Has a tremor |
