# Supplementary material for: Clinical and genotypic analysis in determining dystonia non-motor phenotypic heterogeneity: a UK Biobank study
Source: J Neurol. 2022 Aug 4;269(12):6436–51. doi: 10.1007/s00415-022-11307-4 (PMC9618530; doi:10.1007/s00415-022-11307-4)
Supplement: Supplementary file 2 — Supplementary file2 (DOCX 22 KB) [file 415_2022_11307_MOESM2_ESM.docx]

**Supplementary Table 2: Exclusion codes for UK Biobank cohorts**

|  | | |
| --- | --- | --- |
| **Clinical Terminology** | **Read code** | **ICD-10 code** |
| *Dystonia* |  |  |
| Drug induced dystonia | F1312 | G24.0 |
| *Parkinson’s Disease and secondary parkinsonism* | |  |
| Parkinson's Disease | F12.. | G20 |
| Parkinson's disease NOS | F12z. |  |
| O/E Parkinson gait | 2994. |  |
| O/E - Parkinson posture | 2987. |  |
| O/E - Parkinsonian tremor | 297A. |  |
| Dementia in Parkinson's disease | Eu023 |  |
| FH: Parkinsonism | 129Z. |  |
| Secondary parkinsonism due to other external agents | F12W. | G21.2 |
| Parkinsonism secondary to drugs | F121. | G21.1 |
| Malignant neuroleptic syndrome | F122. | G21.0 |
| Postencephalitic parkinsonism | F123. | G21.3 |
| Vascular parkinsonism | F124. | G21.4 |
| Syphilitic parkinsonism | A94y1 |  |
| Secondary parkinsonism, unspecified | F12X. | G21.9 |
| Secondary parkinsonism |  | G21 |
| Other secondary parkinsonism |  | G21.8 |
| Parkinsonism in diseases EC |  | G22 |
| History of Parkinson's disease | 147F. |  |
| Cerebral degeneration in Parkinson's disease | F11x9 |  |
| *Huntington’s Disease* | | G10 |
| Huntington's chorea | F134. |  |
| Dementia in Huntington's disease | Eu022 |  |
| FH: Huntington’s chorea | 1291. |  |
| *Chorea* | |  |
| Other choreas | F135. | G255 |
| Hemiballismus | F1350 |  |
| Paroxysmal chorea-athetosis | F1351 |  |
| Drug-induced chorea | F1352 | G254 |
| Other choreas NOS | F135z |  |
| *Myoclonus* |  |  |
| Myoclonus | F132. | G253 |
| *Ataxia* | |  |
| Cerebral ataxia | F11y1 |  |
| Cerebellar ataxia NOS | F143. |  |
| Cerebellar ataxia in diseases EC | F144. |  |
| Cerebellar ataxia due to alcoholism | F1440 |  |
| Cerebellar ataxia due to myxoedema | F1441 |  |
| Cerebellar ataxia due to neoplasia | F1442 |  |
| Cerebellar ataxia in disease NOS | F144z |  |
| Congenital nonprogressive ataxia | F145. |  |
| Early onset cerebellar ataxia with hypogonadism | F146. |  |
| Friedreich's ataxia | F140. |  |
| Spinocerebellar disease | F14.. |  |
| Spinocerebellar disease NOS | F14z. |  |
| Other spinocerebellar diseases | F14y. |  |
| Hereditary ataxia |  | G11 |
| *Degenerative diseases of the basal ganglia* | | G23 |
| Other basal ganglia degenerative diseases | F130. |  |
| Dejerine-Thomas syndrome | F1300 |  |
| Hallervorden-Spatz disease | F1301 | G23.0 |
| Striatonigral degeneration | F1302 | G23.2 |
| Parkinsonism with orthostatic hypotension | F1303 |  |
| Progressive supranuclear ophthalmoplegia | F1304 | G23.1 |
| Shy-Drager syndrome | F1305 |  |
| Aicardi Goutieres syndrome | F1306 |  |
| Other basal ganliga degenerative diseases NOS | F130z |  |
| Steele-Richardson-Olszewski syndrome | F24y2 |  |
| Other specified degenerative diseases of basal ganglia |  | G23.8 |
| Degenerative disease of basal ganglia, unspecified |  | G23.9 |
| *Extrapyramidal diseases and movement disorders* |  |  |
| Stiff-man syndrome | F13z1 |  |
| Restless leg syndrome | F13z2 |  |
| Akinetic rigid syndrome | F13z3 |  |
| Hyperekplexia | F13z4 |  |
| Neuroferritinopathy | F13z6 |  |
| Extrapyramidal disease and abnormal movement disorder NOS | F13zz |  |
| Other/unspecified extrapyramidal/abnormal movement disorders | F139. |  |
| Paroxysmal non-kinesigenic dyskinesia | F1390 |  |
| Paroxysmal kinesigenic dyskinesia | F1391 |  |
| *Essential and other specified forms of tremor* |  |  |
| Drug-induced tremor | F1312 | G25.1 |
| Benign essential tremor | F1310 |  |
| *Other cerebral degenerations* |  |  |
| Alzheimer's disease | F110. |  |
| Alzheimer's disease with early onset | F1100 |  |
| Alzheimer's disease with late onset | F1101 |  |
| Pick’s disease | F111. |  |
| Senile degeneration of brain | F112. |  |
| Lewy body disease | F116. |  |
| Frontotemporal degeneration | F118. |  |
| Corticobasal degeneration | F11y2 |  |
| *Hereditary and degenerative diseases of the CNS OS* |  |  |
| Fragile X associated tremor ataxia syndrome | F1y0. |  |
| Hereditary and degenerative diseases of the central nervous system NOS | F1z.. |  |
| *Demyelinating diseases of the central nervous system* |  |  |
| Niemann-Pick disease | C3272 |  |
| Progressive supranuclear palsy | F24y0 |  |
| Wilson's disease | C3510 |  |
| Multiple system atrophy | F174. |  |
| Multiple system atrophy, cerebellar variant | F1740 | G23.3 |
| Multiple system atrophy, Parkinson variant | F1741 |  |
| *Tics/tic disorders* |  | F95 |
| Gilles de la Tourette's disorder | E2723 |  |
| [X]Combined vocal and multiple motor tic disorder [de la Tourette] | Eu952 |  |
| Tic - symptom | 1B24. |  |
| O/E - spasm/tic | 2974. |  |
| Tic disorder unspecified | E2720 |  |
| Transiet childhood tic | E2721 |  |
| Chronic motor tic disorder | E2722 |  |
| Tic NOS | E272z |  |
| [X]Tic disorders | Eu95. |  |
| [X]Transient tic disorder | Eu950 |  |
| [X]Chronic motor or vocal tic disorder | Eu951 |  |
| [X]Involuntary excessive blinking | Eu953 |  |
| [X]Other tic disorders | Eu95y |  |
| [X]Tic disorder, unspecified | Eu95z |  |
| Tics | E272. |  |
| Tics of organic origin | F133. |  |
| *Other degenerative diseases of nervous system, NEC* |  | G31 |
| *Multiple Sclerosis* |  | G35 |
| *Other acute disseminated demyelination* |  | G36 |
| Neuromyelitis optica [Devic] |  | G36.0 |
| Other specified acute disseminated demyelination |  | G36.8 |
| Acute disseminated demyelination, unspecified |  | G36.9 |
| *Other demyelinating diseases of central nervous system* |  | G37 |
| *Infantile cerebral palsy* |  | G80 |
| Spastic cerebral palsy |  | G80.0 |
| Spastic diplegia |  | G80.1 |
| Infantile hemiplegia |  | G80.2 |
| Dyskinetic cerebral palsy |  | G80.3 |
| Other infantile cerebral palsy |  | G80.8 |
| Infantile cerebral palsy, unspecified |  | G80.9 |
| **Key:** EC; Elsewhere Classified, FH; Family History, NEC; Not Elsewhere Classified, NOS; Not Otherwise Specified, O/E; On Examination | | |
