# Supplementary material for: Clinical and genotypic analysis in determining dystonia non-motor phenotypic heterogeneity: a UK Biobank study
Source: J Neurol. 2022 Aug 4;269(12):6436–51. doi: 10.1007/s00415-022-11307-4 (PMC9618530; doi:10.1007/s00415-022-11307-4)
Supplement: Supplementary file 3 — Supplementary file3 (DOCX 32 KB) [file 415_2022_11307_MOESM3_ESM.docx]

| **Table 3: Scores for ICD-10 psychiatric components** | | | | | | | | | | | | | |  |
| --- | --- | --- | --- | --- | --- | --- | --- | --- | --- | --- | --- | --- | --- | --- |
|  | |  | Dystonia | | Cervical Dystonia | | Blepharospasm | | Tremor | | Dystonia, Unspecified | | Control |  |
|  | |  |  | P-value |  | P-value |  | P-value |  | P-value |  | P-value |  |  |
| ICD-10 Diagnoses - Mental and Behavioural Disorders | | | | | | | | | | | | | |  |
| *Schizophrenia, schizotypal and delusional disorders* | | | | | | | | | | | | | |  |
|  | Paranoid Schizophrenia | | 2 (0.1%) | 1 | 0 | - | 0 | - | 0 | - | 2 (1.3%) | 0.242 | 16 (0.06%) |  |
|  | Catatonic Schizophrenia | | 1 (0.06%) | 1 | 0 | - | 1 (0.8%) | 0.190 | 0 | - | 0 | - | 1 (0.004%) |  |
|  | Schizophrenia Unspecified | | 5 (0.3%) | 1 | 1 (0.1%) | 1 | 1 (0.8%) | 1 | 0 | - | 3 (1.9%) | 0.037* | 27 (0.1%) |  |
|  | Delusional Disorder | | 1 (0.06%) | 1 | 0 | - | 0 | - | 0 | - | 1 (0.6%) | 1 | 13 (0.05%) |  |
|  | Schizoaffective Disorder, manic | | 1 (0.06%) | 1 | 0 | - | 0 | - | 0 | - | 1 (0.6%) | 0.545 | 1 (0.004%) |  |
|  | Schizoaffective, Unspecified | | 1 (0.06%) | 1 | 0 | - | 0 | - | 0 | - | 1 (0.6%) | 1 | 6 (0.02%) |  |
| *Mood [affective] disorders* | | | | | | | | | | | | | |  |
|  | Manic episode, unspecified | | 1 (0.06%) | 1 | 0 | - | 0 | - | 0 | - | 1 (0.6%) | 1 | 4 (0.02%) |  |
|  | Bipolar affective disorder, current hypomanic | | 1 (0.06%) | 1 | 0 | - | 0 | - | 1 (0.7%) | 1 | 0 | - | 8 (0.03%) |  |
|  | Bipolar Affective Disorder Current, No Psychosis | | 2 (0.1%) | 1 | 0 | - | 0 | - | 0 | - | 2 (1.3%) | 0.089 | 9 (0.04%) |  |
|  | Bipolar Affective Disorder Current with Mild/Moderate Depression | | 2 (0.1%) | 1 | 0 | - | 0 | - | 1 (0.7%) | 1 | 1 (0.6%) | 0.815 | 2 (0.008%) |  |
|  | Bipolar Affective Current, mixed | | 1 (0.06%) | 1 | 0 | - | 0 | - | 0 | - | 1 (0.6%) | 1 | 4 (0.02%) |  |
|  | Bipolar affective disorder, currently in remission | | 1 (0.06%) | 1 | 0 | - | 0 | - | 0 | - | 1 (0.6%) | 1 | 12 (0.05%) |  |
|  | Bipolar Affective Disorder, Unspecified | | 16 (1.0%) | 0.002** | 2 (0.3%) | 1 | 0 | - | 10 (7.5%) | 1 | 4 (2.6%) | 0.044* | 67 (0.3%) |  |
|  | Moderate Depressive episode | | 5 (0.3%) | 0.883 | 2 (0.3%) | 1 | 0 | - | 2 (1.5%) | 1 | 0 | - | 19 (0.08%) |  |
|  | Severe depressive episode | | 3 (0.2%) | 1 | 2 (0.3%) | 1 | 0 | - | 0 | - | 1 (0.6%) | 1 | 23 (0.09%) |  |
|  | Severe Depressive episode, psychotic | | 2 (0.1%) | 1 | 0 | - | 0 | - | 0 | - | 1 (0.6%) | 1 | 30 (0.1%) |  |
|  | Other Depressive episodes | | 1 (0.06%) | 1 | 1 (0.1%) | 1 | 0 | - | 0 | - | 0 | - | 2 (0.008%) |  |
|  | Depressive episode, unspecified | | 2 (0.1%) | 1 | 0 | - | 0 | - | 0 | - | 1 (0.6%) | 1 | 30 (0.1%) |  |
|  | Recurrent depressive disorder, current episode mild | | 1 (0.06%) | 1 | 0 | - | 0 | - | 1 (0.7%) | 1 | 0 | - | 5 (0.02%) |  |
|  | Recurrent depressive disorder, current episode moderate | | 2 (0.1%) | 1 | 0 | - | 0 | - | 0 | - | 1 (0.6%) | 1 | 31 (0.1%) |  |
|  | Recurrent depressive disorder, unspecified | | 4 (0.3%) | 1 | 0 | - | 0 | - | 0 | - | 4 (2.6%) | 0.005** | 37 (0.2%) |  |
| *Neurotic, stress-related and somatoform disorders* | | | | | | | | | | | | | |  |
|  | Specific (isolated) phobias | | 4 (0.3%) | 1 | 3 (0.4%) | 1 | 0 | - | 1 (0.7%) | 1 | 0 | - | 55 (0.2%) |  |
|  | Other phobic anxiety disorders | | 1 (0.06%) | 1 | 0 | - | 1 (0.8%) | 0.284 | 0 | - | 0 | - | 2 (0.008%) |  |
|  | Panic disorder [episodic paroxysmal anxiety] | | 8 (0.5%) | 1 | 2 (0.3%) | 1 | 1 (0.8%) | 1 | 5 (3.7%) | 1 | 0 | - | 73 (0.3%) |  |
|  | Generalised anxiety disorder | | 2 (0.1%) | 1 | 0 | - | 0 | - | 1 (0.7%) | 1 | 1 (0.6%) | 1 | 22 (0.09%) |  |
|  | Mixed Anxiety and Depressive Disorder | | 12 (0.8%) | 1 | 1 (0.1%) | 1 | 2 (1.5%) | 1 | 5 (3.7%) | 1 | 3 (1.9%) | 1 | 149 (0.6%) |  |
|  | Other specified anxiety disorders | | 1 (0.06%) | 1 | 0 | - | 0 | - | 0 | - | 1 (0.6%) | 1 | 1 (0.004%) |  |
|  | Anxiety Disorder, unspecified | | 44 (2.8%) | 0.197 | 11 (1.4%) | 1 | 5 (3.8%) | 1 | 16 (11.9%) | 0.870 | 12 (7.8%) | 7.32x10^-4^ *** | 418 (1.7%) |  |
|  | Obsessive-compulsive disorder, unspecified | | 2 (0.1%) | 1 | 1 (0.1%) | 1 | 0 | - | 1 (0.7%) | 1 | 0 | - | 10 (0.04%) |  |
|  | Acute stress reaction | | 1 (0.06%) | 1 | 0 | - | 0 | - | 1 (0.7%) | 1 | 0 | - | 10 (0.04%) |  |
|  | Posttraumatic stress disorder | | 1 (0.06%) | 1 | 0 | - | 0 | - | 0 | - | 1 (0.6%) | 1 | 12 (0.05%) |  |
|  | Adjustment Disorder | | 2 (0.1%) | 1 | 0 | - | 1 (0.8%) | 1 | 1 (0.7%) | 1 | 0 | - | 13 (0.05%) |  |
|  | Reaction to severe stress, unspecified | | 2 (0.1%) | 1 | 0 | - | 0 | - | 2 (1.5%) | 1 | 0 | - | 3 (0.01%) |  |
|  | Dissociative Motor Disorder | | 3 (0.2%) | 1 | 0 | - | 0 | - | 0 | - | 2 (1.3%) | 0.074 | 8 (0.03%) |  |
|  | Dissociative [conversion] disorder, unspecified | | 1 (0.06%) | 1 | 0 | - | 0 | - | 0 | - | 1 (0.6%) | 1 | 6 (0.02%) |  |
|  | Hypochondriacal disorder | | 1 (0.06%) | 1 | 0 | - | 0 | - | 0 | - | 1 (0.6%) | 0.815 | 2 (0.008%) |  |
|  | Somatoform autonomic dysfunction | | 1 (0.06%) | 1 | 0 | - | 0 | - | 1 (0.7%) | 1 | 0 | - | 5 (0.02%) |  |
|  | Other Somatoform Disorder | | 5 (0.3%) | 1 | 2 (0.3%) | 1 | 0 | 1 | 3 (2.2%) | 1 | 0 | - | 20 (0.08%) |  |
|  | Neurasthenia | | 1 (0.06%) | 1 | 1 (0.1%) | 1 | 0 | - | 0 | - | 0 | - | 5 (0.02%) |  |
|  | Neurotic disorder, unspecified | | 1 (0.06%) | 1 | 0 | - | 1 (0.8%) | 0.284 | 0 | - | 0 | - | 2 (0.008%) |  |
| *Organic, including symptomatic, mental disorders* | | | | | | | | | | | | | |  |
|  | Dementia in Alzheimer's disease, unspecified | | 3 (0.2%) | 1 | 0 | - | 0 | - | 1 (0.7%) | 1 | 2 (1.3%) | 1 | 41 (0.2%) |  |
|  | Vascular dementia, unspecified | | 1 (0.06%) | 1 | 0 | - | 0 | - | 1 (0.7%) | 1 | 0 | - | 18 (0.07%) |  |
|  | Unspecified dementia | | 4 (0.3%) | 1 | 0 | - | 3 (2.3%) | 0.124 | 0 | - | 1 (0.6%) | 1 | 70 (0.3%) |  |
|  | Delirium, unspecified | | 4 (0.3%) | 1 | 0 | - | 1 (0.8%) | 1 | 1 (0.7%) | 1 | 2 (1.3%) | 1 | 120 (0.5%) |  |
|  | Mild cognitive disorder | | 3 (0.2%) | 1 | 2 (0.3%) | 1 | 0 | - | 1 (0.7%) | 1 | 0 | - | 20 (0.08%) |  |
| *Mental and behavioural disorders due to psychoactive substance use* | | | | | | | | | | | | | |  |
|  | Acute intoxication – Alcohol | | 4 (0.3%) | 1 | 1 (0.1%) | 1 | 0 | - | 3 (2.2%) | 1 | 0 | 1 | 159 (0.6%) |  |
|  | Harmful use - Alcohol | | 14 (0.9%) | 1 | 4 (0.5%) | 1 | 1 (0.8%) | 1 | 5 (3.7%) | 1 | 4 (2.6%) | 1 | 227 (0.9%) |  |
|  | Dependence syndrome – Alcohol | | 9 (0.6%) | 1 | 2 (0.3%) | 1 | 1 (0.8%) | 1 | 3 (2.2%) | 1 | 3 (1.9%) | 1 | 118 (0.5%) |  |
|  | Withdrawal state – Alcohol | | 5 (0.3%) | 1 | 0 | - | 1 (0.8%) | 1 | 2 (1.5%) | 1 | 2 (1.3%) | 1 | 42 (0.2%) |  |
|  | Withdrawal state with delirium - Alcohol | | 1 (0.06%) | 1 | 0 | - | 0 | - | 1 (0.7%) | 1 | 0 | - | 5 (0.02%) |  |
|  | Psychotic disorder due to alcohol use | | 1 (0.06%) | 1 | 0 | - | 0 | - | 1 (0.7%) | 1 | 0 | - | 1 (0.004%) |  |
|  | Harmful use - Cannabinoids | | 1 (0.06%) | 1 | 0 | - | 0 | - | 0 | - | 1 (0.6%) | 1 | 4 (0.02%) |  |
|  | Dependence syndrome – Hypnotics | | 1 (0.06%) | 1 | 0 | - | 0 | - | 0 | - | 1 (0.6%) | 0.815 | 2 (0.008%) |  |
|  | Withdrawal state - Hypnotics | | 1 (0.06%) | 1 | 0 | - | 0 | - | 0 | - | 1 (0.6%) | 0.815 | 2 (0.008%) |  |
|  | Withdrawal state with delirium - hypnotics | | 1 (0.06%) | 1 | 0 | - | 0 | - | 0 | - | 1 (0.6%) | 0.545 | 1 (0.004%) |  |
|  | Unspecified mental and behavioural disorder due to hypnotics use | | 1 (0.06%) | 1 | 0 | - | 0 | - | 0 | - | 1 (0.6%) | 1 | 1 (0.004%) |  |
|  | Acute intoxication - Tobacco | | 1 (0.06%) | 1 | 0 | - | 0 | - | 1 (0.7%) | 1 | 0 | - | 1 (0.004%) |  |
|  | Harmful use – Tobacco | | 79 (5.0%) | 0.526 | 26 (3.4%) | 1 | 9 (6.7%) | 1 | 27 (20.1%) | 1 | 17 (11.0%) | 0.003 ** | 903 (3.7%) |  |
|  | Dependence syndrome - Tobacco | | 4 (0.3%) | 1 | 1 (0.1%) | 1 | 1 (0.8%) | 1 | 1 (0.7%) | 1 | 1 (0.6%) | 1 | 52 (0.2%) |  |
|  | Unspecified mental and behavioural disorder due to tobacco use | | 1 (0.06%) | 1 | 0 | - | 0 | - | 0 | - | 1 (0.6%) | 0.545 | 1 (0.004%) |  |
| *Behavioural syndromes associated with physiological disturbances and physical factors* | | | | | | | | | | | | | |  |
|  | Anorexia nervosa | | 1 (0.06%) | 1 | 1 (0.1%) | 1 | 0 | - | 0 | - | 0 | - | 6 (0.02%) |  |
|  | Bulimia nervosa | | 1 (0.06%) | 1 | 0 | - | 0 | - | 1 (0.7%) | 1 | 0 | - | 1 (0.004%) |  |
|  | Failure of genital response | | 2 (0.1%) | 1 | 0 | - | 1 (0.8%) | 1 | 1 (0.7%) | 1 | 0 | - | 20 (0.08%) |  |
|  | Unspecified sexual dysfunction, not caused by organic disorder or disease | | 1 (0.06%) | 1 | 0 | - | 0 | - | 0 | - | 1 (0.6%) | 0.545 | 1 (0.004%) |  |
| *Disorders of adult personality and behaviour* | | | | | | | | | | | | | |  |
|  | Emotionally unstable personality disorder | | 2 (0.1%) | 1 | 0 | - | 0 | - | 0 | - | 2 (1.3%) | 0.298 | 18 |  |
|  | Anxious [avoidant] personality disorder | | 1 (0.06%) | 1 | 0 | - | 0 | - | 0 | - | 1 (0.6%) | 0.545 | 1 (0.004%) |  |
|  | Personality disorder, unspecified | | 1 (0.06%) | 1 | 0 | - | 0 | - | 0 | - | 1 (0.6%) | 1 | 20 (0.08%) |  |
| Disorders of psychological development | | | | | | | | | | | | | |  |
|  | Asperger's syndrome | | 2 (0.1%) | 1 | 0 | - | 2 (1.5%) | 0.003** | 0 | - | 0 | - | 2 (0.008%) |  |
| Other behavioural and emotional disorders with onset usually occurring in childhood and adolescence | | | | | | | | | | | | | |  |
|  | Stuttering [stammering] | | 2 (0.1%) | 0.725 | 0 | - | 0 | - | 0 | - | 2 (1.3%) | 0.005** | 1 (0.004%) |  |
| Unspecified mental disorder | | | | | | | | | | | | | |  |
|  | Mental disorder, not otherwise specified | | 2 (0.1%) | 1 | 0 | - | 1 (0.8%) | 1 | 0 | - | 1 (0.6%) | 1 | 10 (0.04%) |  |
| **Legend:** P-values are all vs control and are represented as post Bonferroni correction for multiple comparisons, *p<0.05; **p<0.01; ***p<0.001. ICD-10: International Classification of Diseases. | | | | | | | | | | | | | | |
